# Differential expression of the protein kinase A subunits in normal adrenal glands and adrenocortical adenomas

**DOI:** 10.1038/s41598-017-00125-8

**Published:** 2017-03-13

**Authors:** Isabel Weigand, Cristina L. Ronchi, Marthe Rizk-Rabin, Guido Di Dalmazi, Vanessa Wild, Kerstin Bathon, Beatrice Rubin, Davide Calebiro, Felix Beuschlein, Jérôme Bertherat, Martin Fassnacht, Silviu Sbiera

**Affiliations:** 1 Department of Internal Medicine I, Division of Endocrinology and Diabetes, University Hospital, University of Wuerzburg, Wuerzburg, Germany; 2 Comprehensive Cancer Center Mainfranken, University of Wuerzburg, Wuerzburg, Germany; 3 Institut Cochin, INSERM U1016, CNRS UMR810, Department of Endocrinology, Reference Center for Rare Adrenal diseases, Assistance Publique Hôpiteaux de Paris, Hôpital Cochin, Descartes University, Paris, France; 4 Medizinische Klinik and Poliklinik IV, Ludwig-Maximilians University, Munich, Germany; 5 Institute of Pathology, University of Wuerzburg, Wuerzburg, Germany; 6 Institute of Pharmacology and Toxicology and Bioimaging Center, University of Wuerzburg, Wuerzburg, Germany; 7 Endocrinology Unit, Department of Medicine, University of Padua, Padua, Italy; 8 Central Laboratory, University Hospital, University of Wuerzburg, Wuerzburg, Germany

## Abstract

Somatic mutations in protein kinase A catalytic α subunit (*PRKACA*) were found to be causative for 30–40% of cortisol-producing adenomas (CPA) of the adrenal gland, rendering PKA signalling constitutively active. In its resting state, PKA is a stable and inactive heterotetramer, consisting of two catalytic and two regulatory subunits with the latter inhibiting PKA activity. The human genome encodes three different PKA catalytic subunits and four different regulatory subunits that are preferentially expressed in different organs. In normal adrenal glands all regulatory subunits are expressed, while CPA exhibit reduced protein levels of the regulatory subunit IIβ. In this study, we linked for the first time the loss of RIIβ protein levels to the *PRKACA* mutation status and found the down-regulation of RIIβ to arise post-transcriptionally. We further found the PKA subunit expression pattern of different tumours is also present in the zones of the normal adrenal cortex and demonstrate that the different PKA subunits have a differential expression pattern in each zone of the normal adrenal gland, indicating potential specific roles of these subunits in the regulation of different hormones secretion.

## Introduction

ACTH-independent Cushing syndrome is mostly due to cortisol-producing adrenocortical adenomas or carcinomas. Whereas adrenocortical cancer is a very rare tumour^1, 2^, adenomas with autonomous cortisol secretion are frequent^3–5^. With the advent of whole exome sequencing somatic mutations in the gene encoding the catalytic subunit α of protein kinase A (*PRKACA*) has been revealed in approximately 30–40% of CPA, associated with overt Cushing syndrome^6–11^. Also other components of cAMP/PKA signalling were often shown to be either mutated, such as the stimulating G protein (G_as_) which is encoded by the *GNAS* gene or deregulated in different endocrine disorders^12, 13^ indicating the major role played by this pathway in hormonal secretion functions.

Under basal conditions, cAMP-dependent Protein Kinase (PKA) is a stable and inactive heterotetramer, consisting of two catalytic and two regulatory subunits. The human genome encodes three different catalytic subunits (α, β and γ) and four different regulatory subunits (Iα, Iβ, IIα, IIβ) that are each preferentially expressed in different organs^14^. In addition, the protein kinase X was also shown to associate with the PKA regulatory subunits^15^. PKA has two isoforms, referred to as I and II, depending on whether type I or type II regulatory subunits bind the catalytic subunits^16, 17^. Common to all regulatory subunits is the inhibitory role of the activity of the PKA catalytic subunits in the absence of cAMP^18^. PKA signalling is activated when two molecules of cAMP bind each regulatory subunit of the PKA heterotetramer, leading to its dissociation and releasing of the catalytic subunits, which thereby become active. Furthermore, the regulatory subunits can specifically interact with A-kinase anchoring proteins (AKAPs) that target PKA to specific subcellular compartments^19, 20^. This interaction may explain the different expression patterns and roles played by the different PKA isoforms.

Interestingly, the *PRKACA* mutations identified in CPA are situated at the interface between the catalytic subunit and the regulatory subunits^6^. By functional characterization of the initially identified L206R substitution and 199–200 insW insertion, it was shown that the binding of the regulatory subunits to the catalytic subunits is affected by these mutations, rendering PKA constitutively active^21^.

In normal adrenal glands (nAG), as in other endocrine tissues, both type I and II regulatory subunits are expressed^22^, but a detailed analysis had not been performed. Thus, it remains unclear if the expression of the regulatory subunits influences the specific steroidogenic profile of the three zones of the adrenal cortex. Of note, in some CPA, reduced protein levels of the regulatory subunit IIβ have been previously observed^23, 24^. Here we investigate if the *PRKACA* mutations leading to hyperactivity of PKA signalling in CPA are associated with the loss of RIIβ protein levels and demonstrate a clear connection between RIIβ protein levels and the mutation status of *PRKACA*. Furthermore, we describe a comprehensive pattern of PKA subunits expression in the different zones of the adrenal cortex and speculate about the functional consequences of these findings.

## Material and Methods

### Tissue collection

Formalin-fixed paraffin embedded (FFPE) tissues from patients that underwent surgery due to adrenocortical adenomas (n = 103) or carcinomas (n = 33) were consecutively collected at the University Hospitals of Wuerzburg (n = 115), Munich (n = 9) and Padua (n = 12). In addition, 15 FFPE tissues from normal adrenal glands from patients that underwent adrenalectomy due to kidney tumours were consecutively collected at the University Hospital Wuerzburg. Furthermore, fresh frozen tissues of 34 CPA were collected consecutively at the University Hospitals of Wuerzburg (n = 15) and Munich (n = 9), and the Hôpital Cochin Paris (n = 10)^23^ for mRNA and protein analyses. All patients gave informed consent and the study was approved by the Ethics Comities of the University Hospitals of Wuerzburg, Munich and Padua and the Institute Cochin Paris. The *PRKACA* mutation status was determined using exome sequencing (CPA^Cαmut^ n = 8, CPA^CαWT^ n = 13, (endocrine inactive adenomas = EIA n = 13)^6, 11^ or Sanger sequencing of the hotspot around the amino acid L206 (CPA^mut^ n = 15, CPA^WT^ n = 21) (some samples were also part of other studies^6, 10^). In addition, *GNAS* mutation status was determined using exome sequencing (CPA^GNASmut^ n = 7, CPA^GNASWT^ n = 26) or Sanger Sequencing of the hotspot region between amino acids R201 and Q227 (CPA^GNASWT^ n = 5). The methods were carried out in accordance with the approved guidelines. Detailed patient and *PRKACA* mutation data of all CPA are listed in Supplementary Tables S1a and b.

### Patients and clinical annotations

Clinical parameters, such as sex, age at diagnosis, tumour size, and laboratory hormone analysis, and in case of adrenocortical carcinoma (ACC), tumour stage according to the European Network for the Study of Adrenal Tumours (ENSAT) classification^25^, Weiss score, Ki67 index, presence and number of distant metastasis, and detailed follow-up information were collected through the German ACC and the ENSAT Registry (www.ensat.org/registry)^26^. Malignancy and hormonal hypersecretion were defined according to established clinical, biochemical, and morphological criteria. The patient characteristics are listed in the Supplementary Tables S1a,b and S2.

### Chromogenic immunohistochemistry staining

Full FFPE slides were deparaffinised and antigen retrieval was performed in 10 mM citric acid monohydrate buffer (pH 6.5) in a pressure cooker for 13 min. Unspecific binding sites were blocked with 20% human AB serum at room temperature (RT) for 1 h and primary antibodies were incubated in an appropriate dilution concentration at RT for 1 h (PRKACA: BD #610980, 1:1000; PRKAR2B: Sigma #HPA008421, 1:40; PRKAR1A: Novus #29250002, 1:250; PRKAR1B: Abnova #H00005575-M05, 1:100; PRKAR2A: BD #612243, 1:5000 and N-Universal Negative Controls anti-rabbit or anti-mouse: Dako). Signal amplification was achieved by En-Vision System Labelled Polymer-HRP Anti-Rabbit (Dako) for 40 min and developed for 10 min with DAB+ Liquid Kit (Dako). Nuclei were counterstained using Mayer’s hematoxylin for 2 min. Specificities of antibodies were determined by WB. All antibodies gave one specific band at the predicted size.

### Quantitative analysis of PKA subunits immunoreactivity

Chromogenic staining intensities were determined by two independent investigators (I.W. & S.S.) and graded as 0 (negative), 1 (low), 2 (medium) and 3 (high). The proportion of positive tumour cells was calculated for each specimen and scored 0 if 0% were positive, 0.1 if 1–9% were positive, 0.5 if 10–49% were positive and 1 if 50% or more were positive^27, 28^. A semi quantitative H-score was then calculated by multiplying the staining intensity grading score with the proportion score as described previously^29^. Where discrepancies were observed, results were jointly assessed by both investigators and the final score was formed by consensus. The Spearman r for inter-observer agreement for each staining was high (>0.85).

### Fluorescence immunohistochemistry staining

Full FFPE slides were deparaffinised and antigen retrieval was performed as described in the previous chapter. Unspecific binding sites were blocked with 20% human AB serum in TBS and to reduce auto fluorescence, cold water fish skin gelatine (Aurion) was added to a final concentration of 0.1% to the blocking buffer and incubated at RT for 30 min. Primary antibodies were incubated in an appropriate dilution concentration overnight at 4 °C (PRKAR2B: Sigma #HPA008421, 1:30; 58K Golgi protein: GeneTex #GTX26284, 1:50). Fluorescence labelled secondary antibodies against rabbit (goat-anti rabbit-Alexa596; LifeTechnologies #A11072, 1:200) and mouse (goat anti mouse-Alexa488; Dianova #115-545-062, 1:200) were used at RT for 3 h. Vectashield with DAPI (Vector Laboratories #H1200) was used as nuclear counterstain and mounting medium. Optical images were acquired using a Leica SP5 confocal microscope.

### Gene expression analysis

RNA from fresh frozen tissues was isolated using the RNeasy Lipid Tissue Mini Kit (Qiagen) and reverse transcribed with the QuantiTect Reverse Transcription Kit (Qiagen). qRT-PCR was performed using pre-designed Taqman gene expression probes (Thermo Fischer Scientific) for *PRKACA* (Hs00427274_m1) and all PKA regulatory subunits (*PRRKAR1A*: Hs00267597_m1, *PRKAR2A*: Hs00177760_m1; *PRKAR1B*: Hs00406762_m1; *PRKAR2B*: Hs00176966_m1). Endogenously expressed *β*-*actin* (Hs9999903_m1) was used for normalization. 5 ng cDNA were used for each PCR reaction and each sample was analysed in duplicate. Transcripts were amplified using the TaqMan Gene Expression Master Mix (Thermo Fischer), the CFX96 real-time thermocycler (Bio-rad) and Bio-Rad CFX Manager 2.0 software. Cycling conditions were 95 °C for three min followed by 50 cycles of 95 °C for 30 sec, 60 °C for 30 sec, and 72 °C for 30 sec. Using the ΔCT method^30^, the gene expression levels were normalized to those of *β*-*actin*.

### Western blot analysis

20 mg of tumour tissue were shredded in lysis buffer containing 1% Triton-X and protease inhibitors. After sonication, cells were frozen at −80 °C, thawed and protein concentrations were determined using a bicinchoninic acid (BCA) kit (Sigma-Aldrich).

12 µg protein from each sample was loaded on a 10% denaturing gel and proteins were separated by SDS-PAGE^31^. Proteins were transferred by tank blot onto a PVDF Membrane (GE Healthcare) and membranes were blocked in 5% skimmed milk in TBS-Tween at RT for 1 h. Primary antibodies (PRKAR2B: BD #610625, 1:1000 and PRKACA: BD #610980, 1:1000) were incubated over night at 4 °C. Membranes were washed 3 times in TBS-Tween and HRP-labelled secondary antibodies (goat-anti rabbit: Jackson ImmunoResearch Laboratories, #111-035-144 and goat-anti mouse: Jackson ImmunoResearch Laboratories, #115-035-003) were diluted 1:10000 and incubated at RT for 1 h. The protein-antibody complex was visualized by enhanced chemiluminescence using the Amersham ECL Prime reagent (GE Healthcare) and documented on X-ray film (Fuji). Normalization of protein levels was performed by re-probing the blot with an antibody recognizing human GAPDH (Sigma-Aldrich 1:10000). Autoradiographs were scanned, and the quantification of individual bands was performed using the FIJI software^32^.

### Statistical analysis

The Mann-Whitney- U test was used to determine differences between two non-parametric datasets and Kruskal-Wallis test with Dunns post-test were used to determine statistically significant differences between more than two non-parametric datasets. Statistical analyses were performed using GraphPad Prism (version 6.0). P values < 0.05 were considered as statistically significant.

## Results

### RIIβ and RIα protein levels are significantly decreased in Cα-mutated CPA

**Figure 1:**
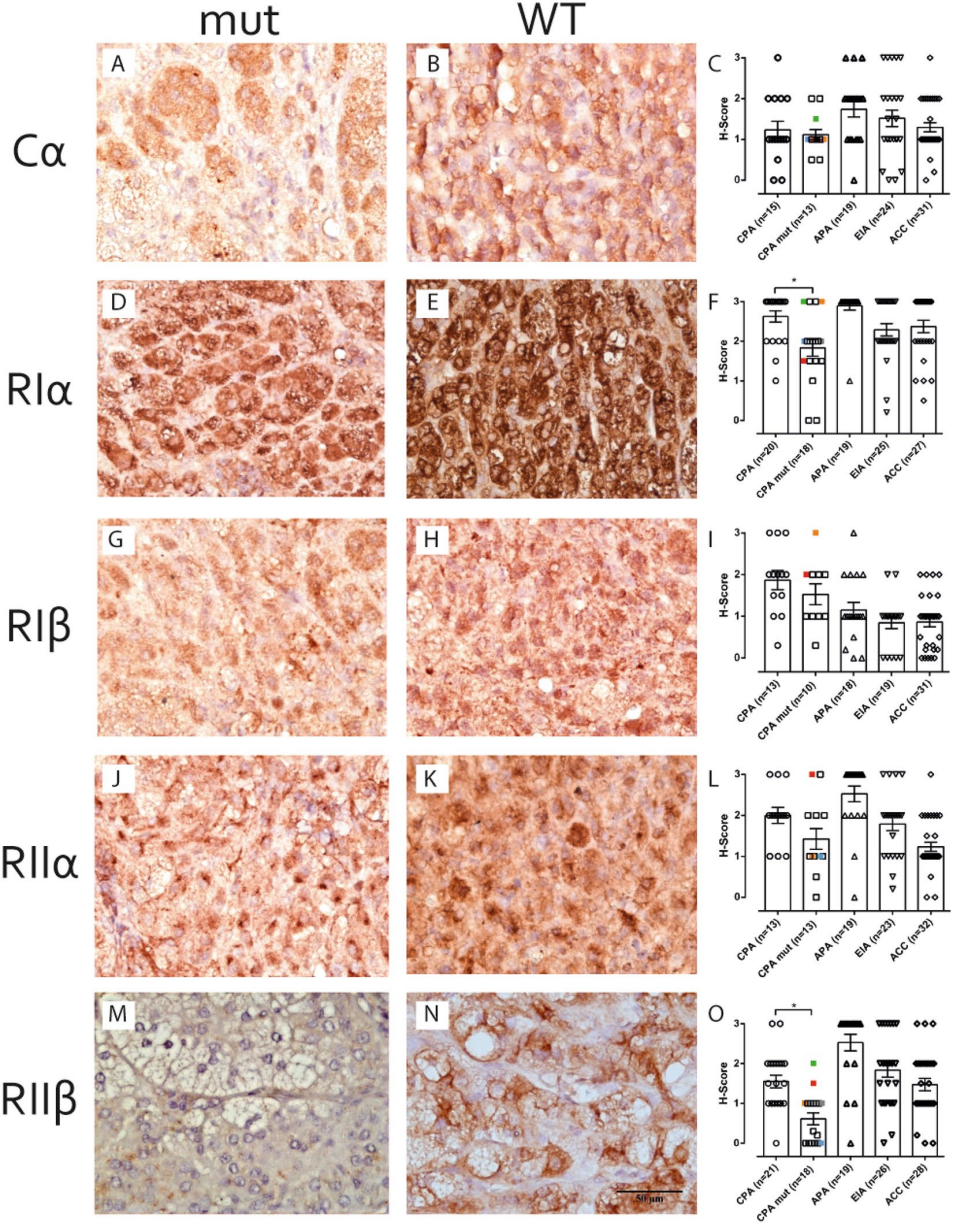
Immunhistochemical staining of the PKA subunits on CPA. Representative immunohistochemical staining for the PKA Cα subunit in CPA ^PRKACAmut^ (**A**) and CPA ^PRKACAWT^ (**B**). An analysis of all stained tissues for Cα is shown in (**C**). Representative immunohistochemical staining for the PKA RIα subunit in CPA^mut^ (**D**) and CPA^WT^ (**E**). An analysis of all stained tissues for RIα is shown in (**F**). Representative immunohistochemical staining for the PKA RIβ subunit in CPA^mut^ (**G**) and CPA^WT^ (**H**). An analysis of all stained tissues for RIβ is shown in (**I**). Representative immunohistochemical staining for the PKA RIIα subunit in CPA^mut^ (**J**) and CPA^WT^ (**K**). An analysis of all stained tissues for RIIα is shown in (**L**). Representative immunohistochemical staining for the PKA RIIβ subunit in CPA^mu^ (**M**) and CPA^WT^ (**N**). An analysis of all stained tissues for RIIβ is shown in (**O**). Mutations, other than the L206R mutation are indicated in different colours. p.200_201insV: blue, p.199_200insW: grey, p.E32V: green, pW197R: orange, p.245_248.del: red. Kruskal-Wallis test with Dunn’s correction for multiple comparisons was performed to determine statistically significant differences between all data sets. *p < 0.05.

Chromogenic immunohistochemistry staining on FFPE tissue from CPA (with mutated (n = 18) and WT (n = 20) *PRKACA*), aldosterone producing adenoma = APA (n = 20), EIA (n = 25) and ACC (n = 33) was performed for all PKA regulatory subunits and the catalytic subunit α. While Cα protein levels were not altered between *PRKACA* ^mut^ CPA (Fig. 1A) and *PRKACA* ^wt^ CPA (Fig. 1B), CPAs in general had lower Cα protein levels compared to the other tumour types (Fig. 1C). RIα had significantly decreased immunoreactivity in *PRKACA* ^mut^ CPA (Fig. 1D) compared to a strong immunoreactivity in *PRKACA* ^wt^ CPA (Fig. 1E). Overall, RIα protein levels were very high in adrenocortical tumours (Fig. 1F).

We did not observe any significant differences in protein levels between *PRKACA* ^wt^ and *PRKACA* ^mut^ CPA for the regulatory subunits IIα and Iβ (mean intensity 1.91 ± 0.67 vs 1.42 ± 0.91, p = 0.13 and 1.78 ± 0.79 vs 1.53 ± 0.79, p = 0.05, respectively) (Fig. 1G–L). However, RIIβ showed no or very weak immunoreactivity in *PRKACA* ^mut^ CPA, while staining intensity was significantly higher in *PRKACA* ^wt^ CPA (0.42 ± 0.48 vs 1.55 ± 0,72, p < 0.05) (Fig. 1M,N). A few *PRKACA* mutated samples showed a strong or normal expression of RIIβ, but they did not harbour the most common, L206R mutation, but other mutations in *PRKACA* (p.E32V: green, p.245_248.del: red, see Fig. 1 legend). Between the different endocrine active adenomas, the strongest staining intensity of RIIβ was observed in APA (2.53 ± 0.90 vs 0.42 ± 0.48 vs 1.55 vs 0.72, p < 0.05) (Fig. 1O).

**Figure 2:**
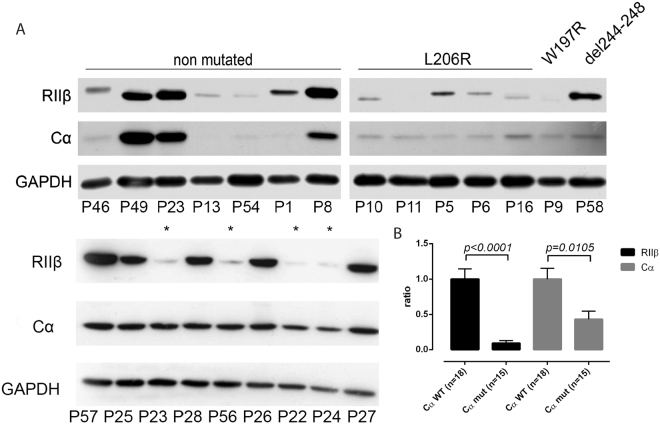
Western blots of cortisol producing adrenocortical adenomas. Western blotting of RIIβ, Cα and GAPDH. The patient ID according to Supplementary Tables S1a and b are given under each band. *PRKACA* mutation status is indicated as such and “*” indicates samples harbouring the L206R mutation in *PRKACA*, as well (**A**). Densiometric analysis of western blots was done using the Fiji software^32^. The quotient of RIIβ/GAPDH and Cα/GAPDH was normalized to the mean of WT RIIβ GAPDH or Cα/GAPDH and this ratio is shown on the y-axis (**B**).

Strongly reduced RIIβ protein levels in Cα-mutated CPA compared to WT CPA were further confirmed by Western blot analysis from three different patient cohorts including 2 cohorts from Germany and one from France (Fig. 2A) (RIIβ to GAPDH ratio in *PRKACA* ^mut^ CPA vs *PRKACA* ^wt^ CPA: 0.08 ± 0.12 vs 0.86 ± 0.53, p < 0.0001) (Fig. 2B). In general, all *PRKACA* mutated samples showed only weak expression of RIIβ. The only exception was the recently detected p.245_248del mutation, which was found in a patient with autonomous cortisol secretion but no overt Cushing syndrome (upper blot). Also Cα protein levels were reduced in mutated compared to WT samples although not to the same extent (Fig. 2B). Since approximately 10% of CPA harbour activating somatic mutations in the *GNAS* gene, leading to increased PKA signalling^7–9^, we investigated if *GNAS* mutations also affect the stability of PKA regulatory subunits. For this purpose, we analysed additionally the CPA ^GNAS mut^ separately from CPA ^PRKACA mut^ and CPA ^PRKACA WT GNAS WT^ both regarding immunohistochemistry and immunoblotting results. However, mutations in the *GNAS* gene did not point to any degradation of the PKA regulatory subunits in CPA (Supplementary Fig. 1).

### mRNA expression of PKA subunits is not significantly altered between Cα-mutated CPA and WT CPA

**Figure 3:**
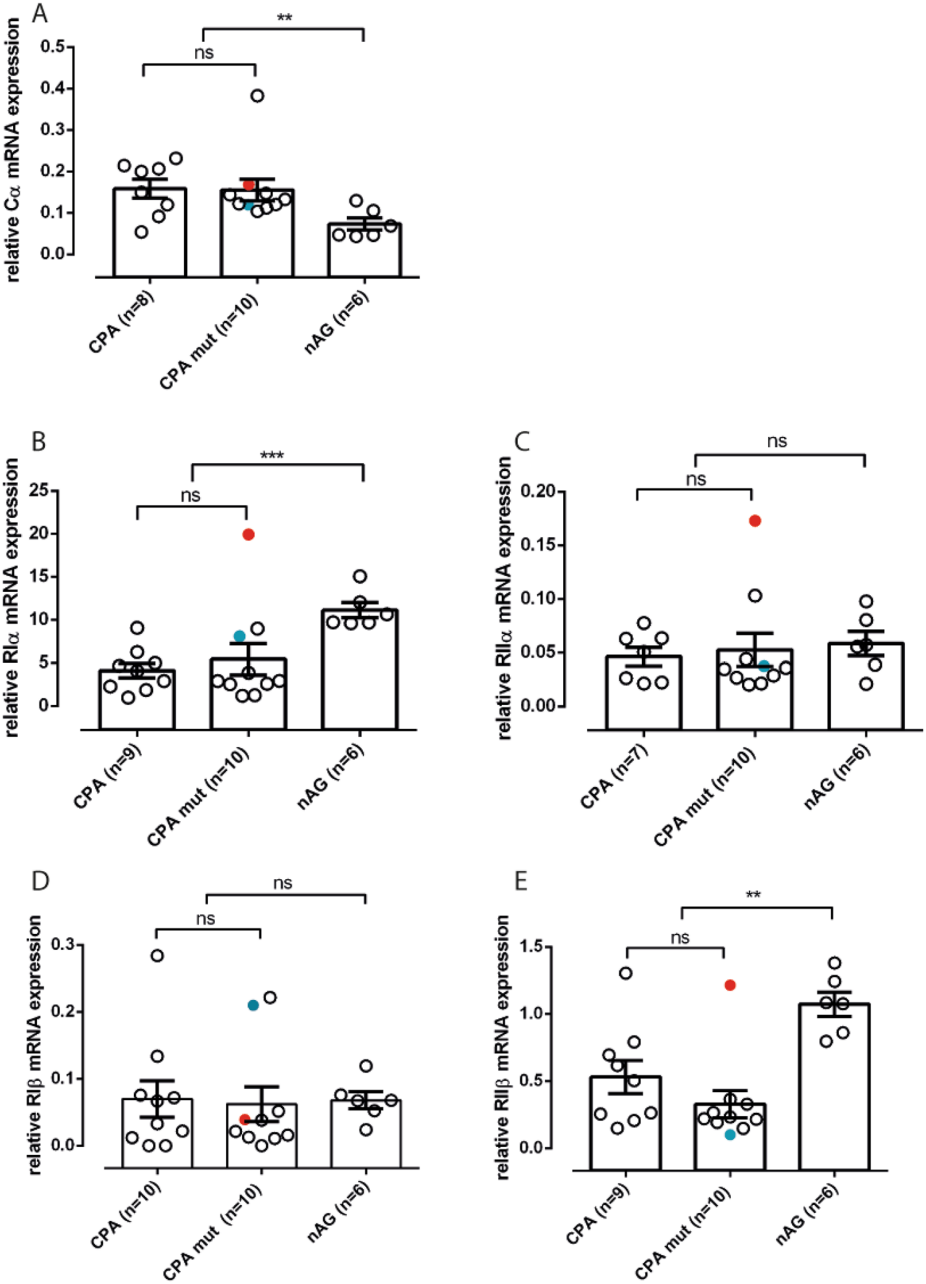
PKA subunits mRNA expression on CPA. Relative PKA subunits mRNA expression (normalized to β-actin using the ΔCT method^30^) for PRKACA (**A**), PRKAR1A (**B**), PRKAR2A (**C**), PRKAR1B (**D**) and PRKAR2B (**E**). The non-parametric Mann-Whitney-U Test was performed to identify statistical differences between CPA (combined CPA *PRKACA* mutated and non mutated) and normal adrenal glands (nAG). Red dot: p.245_248del mutation in *PRKACA*, blue dot: p.W197R *PRKACA* mutation.

To explore if the Cα mutations have an effect on the transcriptional regulation of any PKA subunit, we performed qRT-PCR. mRNA expression levels were not altered between the *PRKACA* ^mut^ and *PRKACA* ^wt^ CPA for all the PKA subunits we tested (Fig. 3), but Cα expression was increased, while RIα and RIIβ expressions were decreased in all CPA compared to nAG (0.16 ± 0.07 vs 0.07 ± 0.04, p = 0.0033; 4.81 ± 4.44 vs 11.12 ± 2.15, p = 0.0003 and 0.42 ± 0.35 vs 1.07 ± 0.22, p = 0.001﻿, Fig. 3A,B,E, respectively).

### RIIβ localizes to the Golgi apparatus in PRKACA^WT^ tumour cells and is not detectable in *PRKACA*^L206R mut^ cells

**Figure 4:**
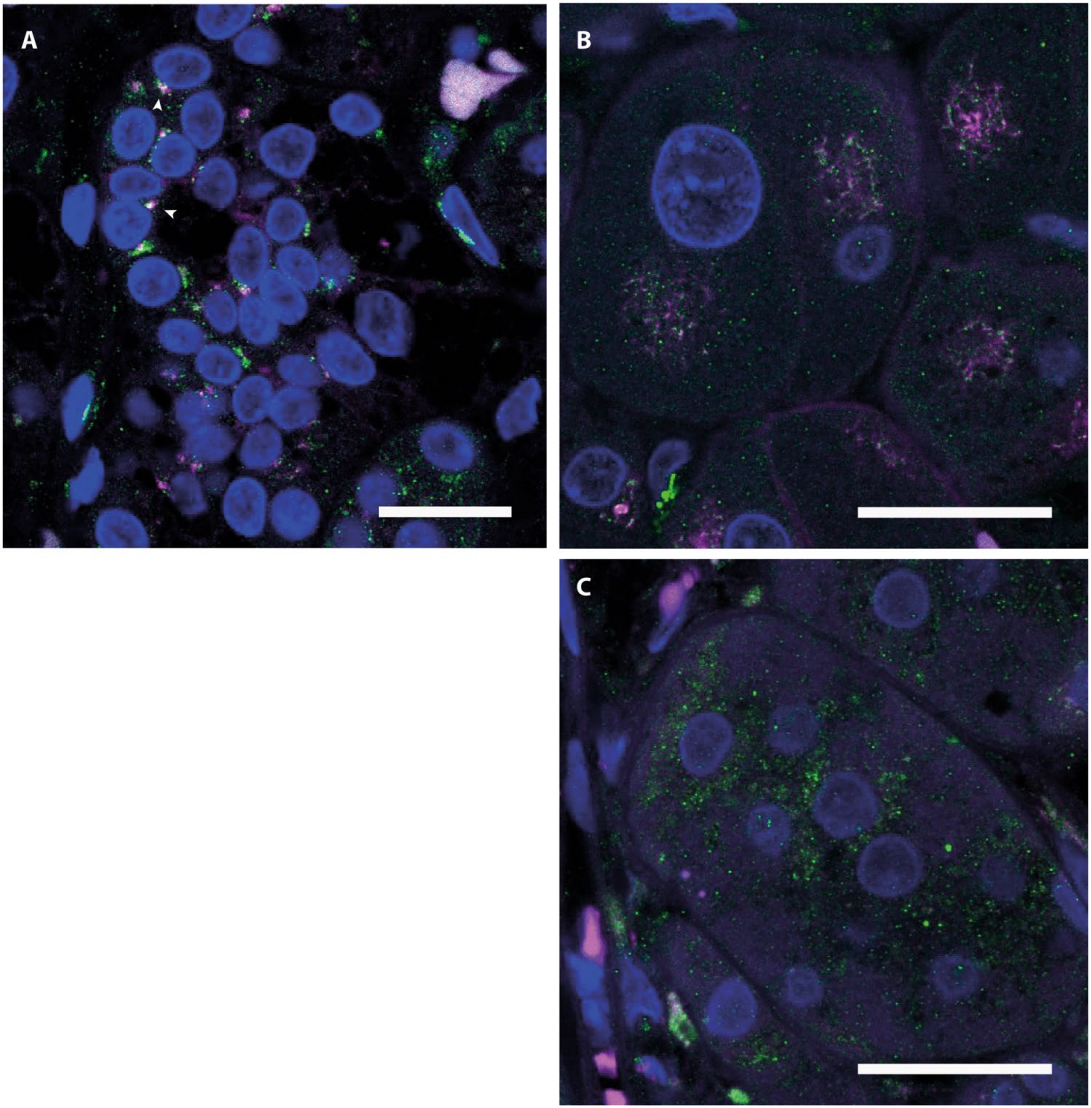
Co-immunofluorescence of RIIβ and the Golgi apparatus. Representative co-immunofluorescence of RIIβ (magenta) and the Golgi marker 58K protein (green) in a PRKACA^WT^ tumour (**A**,**B**) and in an PRKACA^L206R mut^ tumour (**C**). The overview shows accumulation of RIIβ in localized areas close to the nucleus and the overlay with the Golgi apparatus marker reveals co-localization (white, arrowheads). A higher magnification of the same tumour shows the close proximity of RIIβ and the Golgi apparatus (**B**), while the RIIβ staining is absent in the L206R mutated tumour (**C**). Scale bar 20 µm (**A**) and 10 µm (**B**,**C**).

In order to investigate, a possible connection between reduced RIIβ protein levels in *PRKACA* mutated tumours and its subcellular localization in these tumours, we performed simultaneous fluorescence immunohistochemistry of RIIβ and the Golgi apparatus in a subset of 10 CPA (6 WT and 4 mutated). Co-staining of RIIβ and several different organelle markers in adrenocortical tumour cells NCI-H295R, revealed a co-localization of RIIβ with the Golgi apparatus but not with the endoplasmic reticulum, mitochondria or lysosomes (Supplementary Material & Methods; Supplementary Fig. 2). And indeed, in our stained *PRKACA* ^WT^ CPA tissues, RIIβ had a very localized distribution, accumulating in small spots close to the nucleus (Fig. 4A). Golgi apparatus staining showed a very similar pattern and the overlay revealed a close proximity of RIIβ and Golgi apparatus (Fig. 4A,B). In *PRKACA* ^L206Rmut^ CPA, RIIβ staining was not detectable and there was no indication that it changed location in these cells (Fig. 4C).

### The PKA subunits are differentially expressed in normal adrenal glands

**Figure 5:**
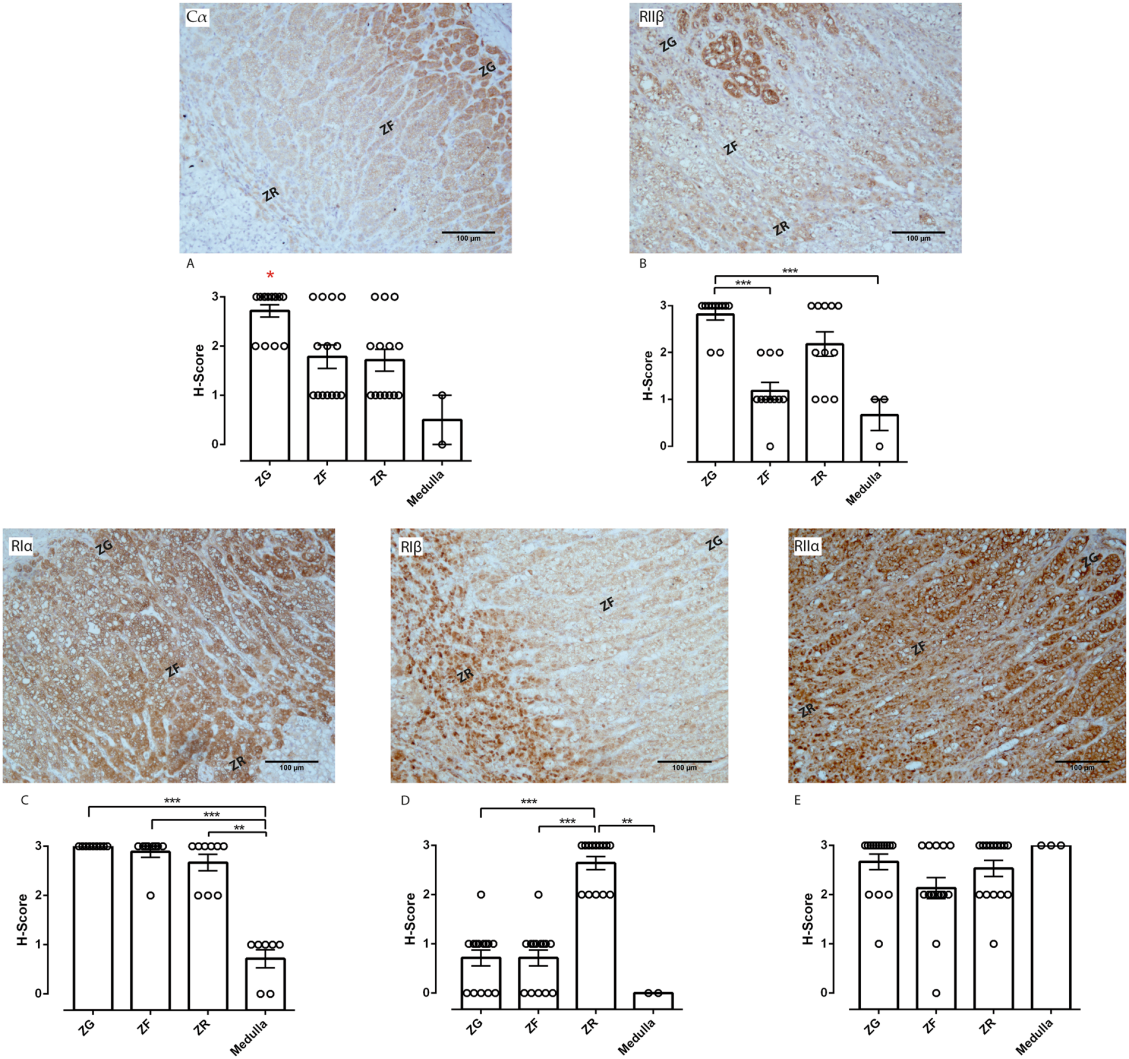
Immunohistochemical staining of the PKA subunits in normal adrenal glands. Representative immunohistochemical staining of the PKA subunits in the different zones of the adult normal adrenal gland and analysis of staining intensities of the different zones of the adrenal cortex and the medulla. Cα is strongly expressed in the *Zona glomerulosa* (**A**), RIIβ is strongly expressed in the *Zona glomerulosa* and *Zona reticularis* (**B**), RIα is strongly expressed in all zones of the adrenal cortex (**C**), RIβ has a strong expression in the *Zona reticularis* (**D**) and RIIα is expressed in the whole adrenal gland including the medulla (**E**). ZG: *Zona glomerulosa*, ZF: *Zona fasciculata*, ZR: *Zona reticularis*. Kruskal-Wallis test with Dunn’s correction for multiple comparisons was performed to determine statistically significant differences between all data sets. *p < 0.05, **p < 0.01, ***p < 0.001.

The above mentioned observations that the different PKA subunits have different expression patterns between the tumours with different hormonal pattern suggest some relation between PKA subunit expression and steroid secretion. Therefore, we analysed expression levels of these subunits in the different zones of the adrenal cortex and the medulla. Cα protein levels were highest in the *zona glomerulosa*, but it was also moderately strong expressed in the other two zones of the cortex and was almost absent in the medulla (Fig. 5). In general, expression of all subunits was rather low in the medulla with exception of RIIα, which in contrast to all other subunits was almost ubiquitously strong throughout the adrenal gland (RIα: 0.71 ± 0.49; RIIα: 3.0 ± 0.0; RIβ: 0.0 ± 0.0; RIIβ: 0.67 ± 0.58; Cα: 0.50 ± 0.71) (Fig. 5E). The *zona glomerulosa* seems to be characterized by low expression of RIβ, whereas all other subunits are rather highly expressed (RIα: 3.0 ± 0.0; RIIα: 2.67 ± 0.62; RIβ: 0.71 ± 0.61; RIIβ: 2.82 ± 0.40; Cα: 2.71 ± 0.47) (Fig. 5). The pattern in the cortisol-producing *zona fasciculata* is strikingly similar with one exception, the low expression of RIIβ (RIα: 2.89 ± 0.33; RIIα: 2.13 ± 0.83; RIβ: 0.71 ± 0.61; RIIβ: 1.18 ± 0.60; Cα: 1.79 ± 0.89) (Fig. 5B). Thus, the two β subunits seem to be negatively correlated with secretion of aldosterone and cortisol, a pattern which can also be partly found in the aldosterone- and cortisol-producing adenomas. Interestingly, the *zona reticularis*, which produces quantitatively the highest amount of steroid hormones in the circulating blood, mainly precursors and androgen, is characterized by high expression of all four regulatory subunits (RIα: 2.67 ± 0.5; RIIα 2.53 ± 0.64; RIβ: 2.64 ± 0.5; RIIβ: 2.18 ± 0.87; Cα: 1.71 ± 0.83) (Fig. 5).

## Discussion

### Decreased protein levels of PKA RIIβ and RIα subunits in cortisol producing adenomas

In 2008 two studies had shown that protein expression of the RIIβ subunit of PKA is markedly reduced in CPA compared to other adrenocortical tumours, while no such differences were observed regarding the expression of the other regulatory subunits^23, 24^. There were some discrepancies between the two studies, with Mantovani *et al*. demonstrating that a loss of RIIβ results in a compensatory increase of RIα protein levels in Y1 cells, while Vincent-Dejean *et al*. failed to observe this phenomenon in tumour samples. Furthermore, the latter found decreased RIIβ protein levels only in a subpopulation of CPA with increased PKA activity. This discrepancy came as a surprise, as the compensatory up-regulation of one regulatory PKA subunit in response to down-regulation of the other has been shown in several systems^33, 34^. One study demonstrated that also mutations in the regulatory subunit RIα, leading to its decreased expression, induced the compensatory mechanism leading to an increase of other regulatory subunits^34^. The down-regulation of RIIβ in CPA could not be explained for some time. Recently, we could prove that mutations in the Cα subunit decrease the binding potential of regulatory subunits^21^ and already many years ago it was demonstrated, that unbound regulatory subunits are normally degraded in the proteasome in a ubiquitin-dependent manner^35^. Following the discovery of mutations in the gene encoding for the catalytic subunit α of PKA as the underlying cause of CPA tumourigenesis^6^ we hypothesized that these mutations might be causative for the decreased RIIβ protein expression in these tumours. Therefore, we assessed the protein levels of PKA subunits by both immunohistochemistry and immunoblotting in a large series of adrenocortical tumours with known *PRKACA* mutation status, including 10 samples that had already been included in the study by Vincent-Dejean *et al*.^23^.

Since reduced RIIβ protein levels were found in CPA in general^24^ but were particularly pronounced in a subpopulation of CPA with high PKA activity^23^, we hypothesized, that this subpopulation is the *PRKACA* mutated CPA group. This hypothesis was confirmed by our immunohistochemistry results where CPA with mutations in the *PRKACA* gene showed reduced RIIβ protein levels compared to *PRKACA* ^wt^ CPA. We also observed significantly reduced RIα protein levels in *PRKACA* ^mut^ compared to *PRKACA* ^wt^ CPA but no significant differences concerning the other two regulatory subunits or the catalytic subunit itself. One possible explanation for this down-regulation could be the general differences in abundance of these two regulatory subunits in the adrenal cortex, so the degradation of unbound regulatory subunits failing to bind the mutated catalytic subunits may be stronger in the case of the subunits with higher adrenal expression.

Surprisingly, the only *PRKACA* ^wt^ CPA that lacked immunoreactivity for RIIβ in immunohistochemistry was from a patient with autonomous cortisol secretion and did not reveal any somatic mutations affecting the cAMP/PKA signalling pathway. Interestingly, in this sample, by whole exome sequencing, we could identify a germline genomic variant in the *GNAS* gene (encoding the alpha subunit of the stimulatory G protein (Gs) and involved in cAMP regulation) (p.R600G, SNP: rs74897360)^11^, however, the clinical significance of this SNP has never been investigated before.

Mantovani *et al*. found decreased RIIβ protein levels in CPA compared to ACC^24^. In our series only the *PRKACA* ^mut^ CPA had significantly decreased RIIβ levels compared to ACC but not WT CPA. Neither did we observe any significant differences between the other PKA subunits between any of the adrenocortical adenomas subgroups, nor when we compared them with adrenocortical carcinomas. This previous study also showed that in the low RIIβ group there was also a significant reduction in RIIα protein and a tendency for reduced Cα and RIα subunits protein expression. Although we did not investigate RIIα with immunoblotting, we did not observe reduced RIIα protein levels in *PRKACA* ^mut^ CPA by immunohistochemistry. However, what we could show was that the Cα and RIα protein levels were both significantly reduced in *PRKACA* ^mut^ compared to *PRKACA* ^wt^ CPA. The discrepancy between these results and the fact that immunohistochemistry did not reveal a similar reduction for the Cα could arise from partly different samples and also from the two methods used. But also other groups found a reduced expression of the mutant Cα protein^8^ in the tumours.

In contrast to the samples carrying the frequent L206R *PRKACA* mutations, decrease of RIIβ protein levels was observed in only some of the other mutated tumours. Highest RIIβ expression was observed in the sample carrying the p.E32V mutation^11^, which lies outside the interface of regulatory and catalytic subunits and does maybe not influence the formation of a stable PKA holoenzyme. Unchanged RIIβ protein expression in western blot analysis was also found in the sample carrying the p.245_248.del mutation^11^, however, this tumour is from a patient without signs of clinical Cushing Syndrome, suggesting that the effects of this mutation are less severe.

As at mRNA level, we did not observe any downregulation of RIα and RIIβ in *PRKACA* ^mut^ samples compared to *PRKACA* ^wt^ CPA, therefore a post-translational regulation and/or degradation is a more likely explanation for the decrease in protein levels. In 2011, it was shown that the E3 ligase Praja2 interacts with the regulatory subunits Iα, IIα and IIβ, tagging them for proteasomal degradation in order to prolong the activated PKA signalling^36^. However, our results indicate a more restricted mechanism of degradation, especially for RIIβ in *PRKACA* ^mut^ samples, since RIIβ protein levels were completely absent in most samples. This degradation could be mediated by a RIIβ-specific A-kinase anchoring protein (AKAP), that modifies either its subcellular localization or tags it for proteasomal/lysosomal degradation. Indicative for such a degradation tagging would be the change of RIIβ protein localization between *PRKACA* ^*wt*^ and *PRKACA* ^mut^ samples towards a more proteasomal/lysosomal localization. As we hypothesized that the *PRKACA* mutations possibly changes RIIβ subcellular localization we checked if RIIβ is also co-localized with the golgi apparatus in tissues derived from *PRKACA* ^wt^ and *PRKACA* ^L206Rmut^ CPA or if this localization changes in the mutated samples. A co-localization of RIIβ with the Golgi apparatus has already been described in different cell types^37, 38^. This hypothesis proved not to be true so we don’t have yet a conclusive answer for the reasons leading to low levels of RIIβ in *PRKACA* ^L206Rmut^ CPA. Now, additional functional studies would be necessary to answer this question but they are beyond the scope of this publication.

Interestingly, other groups investigating the expression of the PKA subunits in other endocrine tissues such as thyroid and thyroid derived tumours^39^ found exactly the opposite, no to low levels of RIIβ and RIα in normal thyroid tissue but both subunits had increased levels in both benign and malignant thyroid tumours^39^ showing that PKA function in endocrine organs is very variable.

### Possible correlation between expression of different PKA subunits and hormone secretion pattern in normal adrenal glands

A differential expression of several PKA isoforms in different tissues or subcellular compartments has already been described^14, 38^. Here we have demonstrated further, that some PKA subunits are not only organ specific, but that their expression is different between the different functional zones of the adrenal cortex, indicating possible specific roles in the secretion of hormones. The catalytic subunit α is most abundantly expressed in the *zona glomerulosa*, the zone of the adrenal cortex that secretes mainly mineralcorticoids. However, the strong expression of Cα seems to be compensated by a strong expression of all regulatory subunits, with the exception of RIβ, in this zone, suggesting a high level of regulation through formation of an inactive PKA heterotetramer. RIβ showed the most limited expression pattern in the adrenal gland being restricted to the *zona reticularis*, responsible for sex hormones secretion. Therefore, it could be involved in the regulation of these hormones. RIIα was the only subunit that showed expression in all zones of the adrenal cortex and additionally in the adrenal medulla, a part of the adrenal gland of neuroendocrine origin that secretes catecholamines. RIIβ, although considered to be one of the main regulatory subunits in the adrenal glands, showed only a weak to medium immunoreactivity in the *zona fasciculata*, which produces mainly cortisol but was abundant in the *zona glomerulosa* and *zona reticularis*. Together with the fact that low RIIβ expression is associated with an increase in cortisol production in a subset of adrenocortical adenomas, this suggests that at least in the adrenal cortex this subunit is responsible for the negative regulation of cortisol production.

In conclusion, our study shows a direct link between *PRKACA* mutations and reduced protein levels of two regulatory subunits of PKA in a sub-group of CPA and that specific expression overlapping the different functional zones of the adrenal cortex, could indicate distinct roles of the different PKA regulatory subunits in the secretion of different hormones.

## Electronic supplementary material

Supplementary information
