# Supplementary material for: Differential expression of the protein kinase A subunits in normal adrenal glands and adrenocortical adenomas
Source: Sci Rep. 2017 Mar 13;7:49. doi: 10.1038/s41598-017-00125-8 (PMC5427838; doi:10.1038/s41598-017-00125-8)
Supplement: Supplementary file 1 — Supplementary information [file 41598_2017_125_MOESM1_ESM.pdf]

Supplementary information

Differential expression of the protein kinase A subunits in normal adrenal glands and adrenocortical adenomas

Isabel Weigand[1], Cristina L. Ronchi[2], Marthe Rizk-Rabin[3], Guido Di Dalmazi[4], Vanessa Wild[5], Kerstin Bathon[6], Beatrice Rubin[7], Davide Calebiro[6], Felix Beuschlein[4], Jérôme Bertherat[3], Martin Fassnacht[1,2,8] & Silviu Sbiera[1,2]

[1]Department of Internal Medicine I, Division of Endocrinology and Diabetes, University Hospital, University of Wuerzburg, Wuerzburg, Germany; [2]Comprehensive Cancer Center Mainfranken, University of Wuerzburg, Wuerzburg, Germany; [3] Institut Cochin, INSERM U1016, CNRS UMR810, Descartes University; Department of Endocrinology, Reference Center for Rare Adrenal diseases, Assistance Publique Hôpiteaux de Paris, Hôpital Cochin, Paris, France; [4]Medizinische Klinik and Poliklinik IV, Ludwig-Maximilians University, Munich, Germany; [5] Institute of Pathology, University of Wuerzburg, Wuerzburg, Germany; [6] University of Wuerzburg, Institute of Pharmacology and Toxicology and Bioimaging Center, Wuerzburg, Germany; [7]Endocrinology Unit, Department of Medicine, University of Padua, Padua, Italy; [8]Central Laboratory, University Hospital, University of Wuerzburg, Wuerzburg, Germany

Corresponding author:

Silviu Sbiera

Division of Endocrinology and Diabetes, Department of Internal Medicine I

University Hospital, University of Wuerzburg,

Oberduerrbacherstr. 6

97080 Wuerzburg, Germany

Tel: +49(0)931-20139702

Fax: +49(0)931-201639702

Email: Sbiera_S@ukw.de

Adrenocortical cell line culture conditions, fixation & immunofluorescence

NCI-H295R cells were grown as monolayers in DMEM/F12 (1:1) medium (Gibco) supplemented with insulin, transferrin and selenium (Gibco) and 2.5% Nu-serum (Corning) at 37°C and 5% CO_2 in chamber slides. Fixation occurred with 4% PFA in PBS at RT for 8 min and was followed by permeabilization with 0.5% Triton-X in PBS. Primary antibodies were incubated in an appropriate dilution concentration for 1h at RT (Golgi marker giantin (abcam #ab37266, 1:50); ER marker calnexin (Novus #NB300-518, 1:50); mitochondria marker MTCO2 (abcam #ab3298, 1:50) and lysosome marker Lamp1 (abcam #ab24170, 1:50)). Fluorescence labeled secondary antibodies against rabbit (goat-anti rabbit-Alexa596; LifeTechnologies #A11072, 1:200) and mouse (goat anti mouse-Alexa488; Dianova #115-545-062, 1:200) were used at RT for 1h. Vectashield with DAPI (Vector Laboratories #H1200) was used as nuclear counterstain and mounting medium. Optical images were acquired using a Leica SP5 confocal microscope.

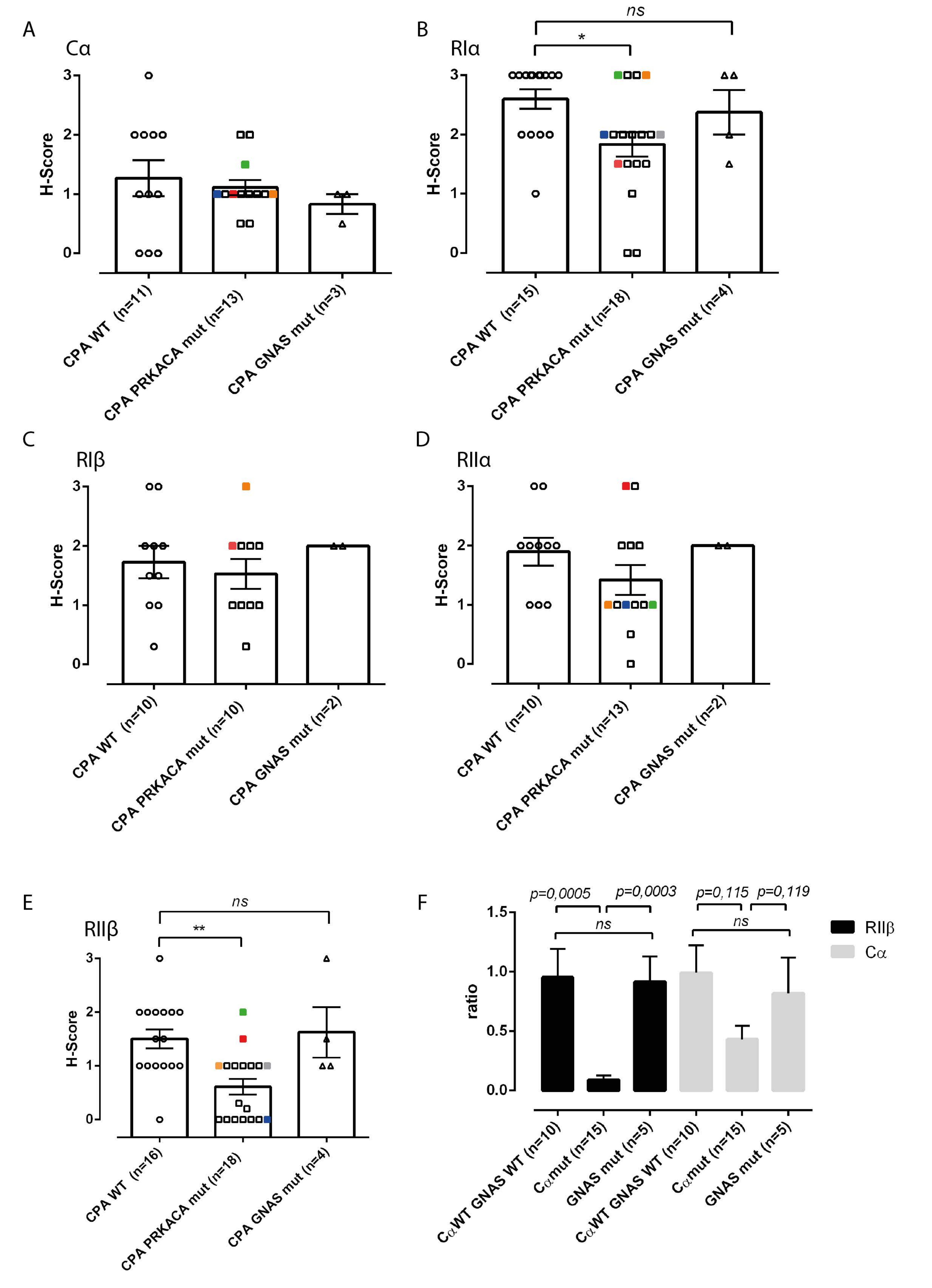

Figure S1: Immunhistochemical staining of the PKA Cα subunit in CPA PRKACA^{WT} GNAS^{WT}, CPA PRKACA^{mut} and CPA GNAS^{mut} (**A**), the PKA RIα subunit in CPA WT, CPA PRKACA^{mut} and CPA GNAS^{mut} (**B**), the PKA RIβ subunit in CPA WT, CPA PRKACA^{mut} and CPA GNAS^{mut} (**C**), the PKA RIIα subunit in CPA WT, CPA PRKACA^{mut} and CPA GNAS^{mut} (**D**) and the PKA RIIβ subunit in CPA WT, CPA PRKACA^{mut} and CPA GNAS^{mut} (**E**). Immunoblot analysis of the PKA subunits RIIβ and Cα in CPA WT, CPA PRKACA^{mut} and CPA GNAS^{mut} (**F**). PRKACA mutations other than L206R are indicated in different colours. p.200_201insV: blue, p.199_200insW: grey, p.E32V: green, p.W197R: orange, p.245_248.del: red. Kruskal-Wallis test with Dunn's correction for multiple comparisons was performed to determine statistically significant differences between all data sets (A-E). Mann-Whitney-U Test was performed to determine statistically significant differences between two independent data sets (F). *p<0.05, **p<0.01

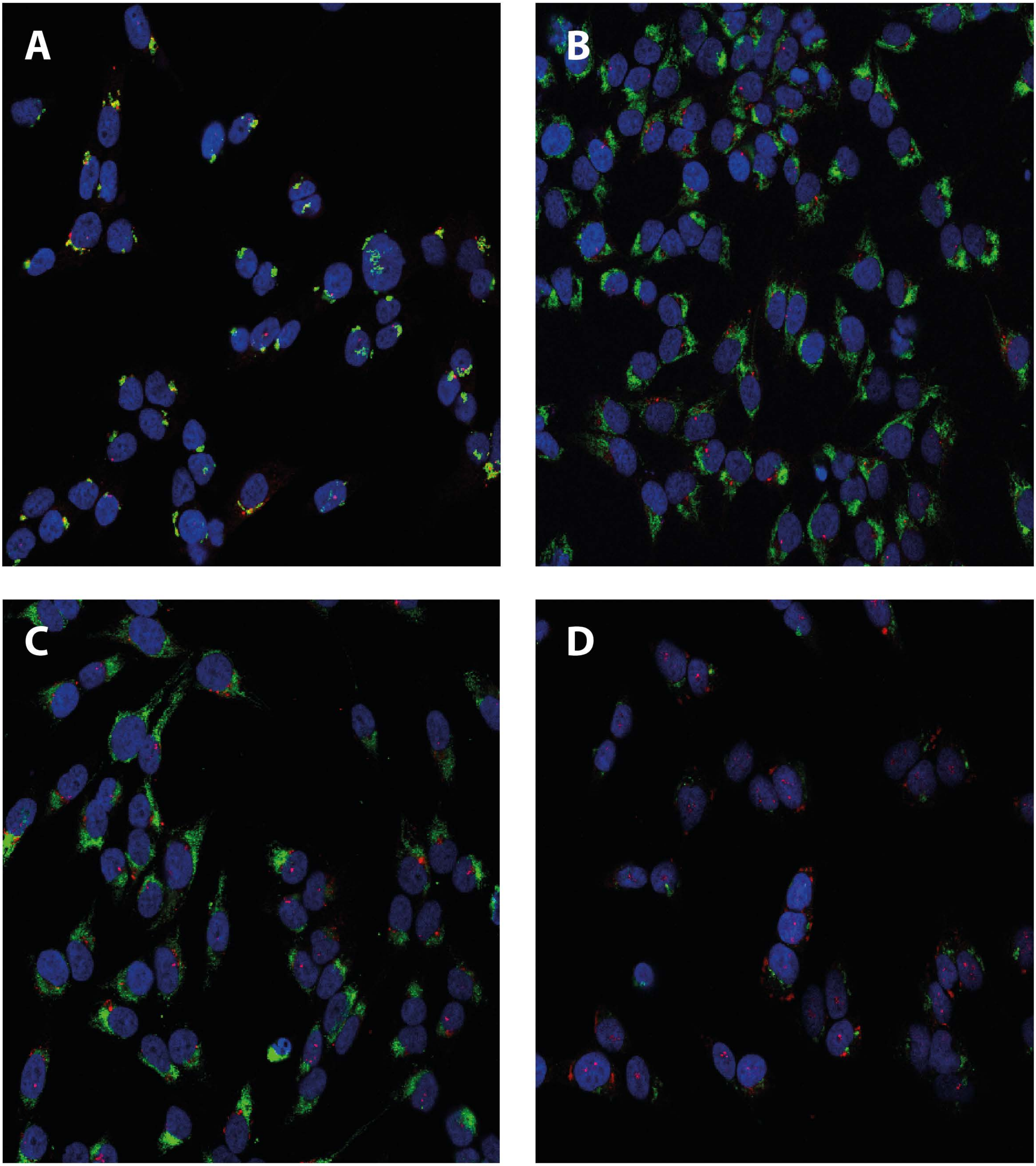

Figure S2: In the adrenocortical carcinoma cell line NCI-H295R a co-immunofluorescence of RIIβ (red) with the golgi marker giantin (green) revealed a co-localisation of both proteins (A), but not with the endoplasmatic reticulum marker calnexin (green) (B), the mitochondria marker MTCO2 (green) (C), or the lysosome marker LAMP1 (red) (D).

Supplementary Table S1a: Clinical characteristics of all CPA *PRKACA*^{mut} used. Median age and mean values are indicated after each tumour group. Tumour sizes are given in cm, Cortisol post dexamethasone results are given in µg/dl.

#	Diagnose	*PRKACA* mutation	Age	Sex	Tumor size	Cort post Dexa
P5	overt Cushing	L206R	25	F	4	29,2
P6	overt Cushing	L206R	29	F	3,3	na
P7	overt Cushing	L206R	61	F	2,9	18,2
P9	overt Cushing	W197R	45	F	na	na
P10	overt Cushing	L206R	43	F	3	na
P11	overt Cushing	L206R	42	F	1,8	na
P12	overt Cushing	L206R	60	F	3,5	na
P16	overt Cushing	L206R	41	F	4	22,4
P21	overt Cushing	199_200insW	48	F	2,2	14,8
P22	overt Cushing	L206R	34	F	3	26,4
P23	overt Cushing	L206R	47	F	3	21
P24	overt Cushing	L206R	49	F	3,6	na
P29	overt Cushing	L206R	40	F	2,8	na
P30	overt Cushing	insV	48	F	2,5	20
P33	overt Cushing	E32V	38	F	2,3	12,6
P37	overt Cushing	L206R	55	F	3	na
P38	overt Cushing	L206R	57	F	4	na
P41	overt Cushing	L206R	37	F	3,5	>5
P42	overt Cushing	L206R	38	F	3	na
P45	overt Cushing	L206R	38	F	3	19,6
P55	autonomous cortisol secretion	L206R	52	M	3	16,7
P56	autonomous cortisol secretion	L206R	37	F	2,6	12,6
P58	autonomous cortisol secretion	del445-448	34	M	4	5,5
			42	22F/ 2M	3,1	18,3

Supplementary Table S1b: Clinical characteristics of all CPA *PRKACA*^{WT} used. Median age and mean values are indicated after each tumour group. Tumour sizes are given in cm, Cortisol post dexamethasone results are given in µg/dl.

#	Diagnose	*PRKACA* mutation	*GNAS* mutation	Age	Sex	Tumor size	Cort post Dexa
P1	overt Cushing	WT	WT	55	F	5,2	10,9
P2	overt Cushing	WT	WT	44	M	3,5	33,9
P3	overt Cushing	WT	WT	35	M	3,3	5,6
P4	overt Cushing	WT	WT	46	F	3	na
P8	overt Cushing	WT	WT	31	F	3,2	na
P13	overt Cushing	WT	WT	55	F	6,5	15,7
P14	overt Cushing	WT	WT	71	M	11	14,4
P15	overt Cushing	WT	WT	46	F	na	na

**P17**	**overt Cushing**	**WT**	**WT**	**36**	**F**	**3,4**	**15**
**P18**	**overt Cushing**	**WT**	**WT**	**32**	**F**	**na**	**14**
**P19**	**overt Cushing**	**WT**	**WT**	**67**	**F**	**3**	**11,6**
**P20**	**overt Cushing**	**WT**	**WT**	**44**	**F**	**4**	**3,2**
**P25**	**overt Cushing**	**WT**	**WT**	**51**	**F**	**na**	**na**
**P26**	**overt Cushing**	**WT**	**R201C**	**35**	**F**	**3,4**	**15,1**
**P27**	**overt Cushing**	**WT**	**WT**	**71**	**F**	**2,3**	**na**
**P28**	**overt Cushing**	**WT**	**na**	**40**	**F**	**4**	**na**
**P31**	**overt Cushing**	**WT**	**WT**	**19**	**F**	**3**	**30,2**
**P32**	**overt Cushing**	**WT**	**R201H**	**23**	**F**	**na**	**na**
**P34**	**overt Cushing**	**WT**	**WT**	**48**	**F**	**3**	**na**
**P36**	**overt Cushing**	**WT**	**na**	**70**	**F**	**2,5**	**>5**
**P39**	**overt Cushing**	**WT**	**WT**	**50**	**F**	**5,2**	**na**
**P40**	**overt Cushing**	**WT**	**na**	**41**	**F**	**2,5**	**na**
**P43**	**overt Cushing**	**WT**	**R201**	**51**	**F**	**4**	**>5**
**P44**	**overt Cushing**	**WT**	**R201**	**37**	**F**	**5,5**	**>5**
**P46**	**autonomous cortisol secretion**	**WT**	**R201C**	**73**	**F**	**3**	**na**
**P47**	**autonomous cortisol secretion**	**WT**	**WT**	**51**	**F**	**8**	**3,4**
**P48**	**autonomous cortisol secretion**	**WT**	**R201H**	**29**	**F**	**2**	**14**
**P49**	**autonomous cortisol secretion**	**WT**	**WT**	**35**	**F**	**3**	**8,1**
**P50**	**autonomous cortisol secretion**	**WT**	**WT**	**58**	**F**	**3,2**	**14,6**
**P51**	**autonomous cortisol secretion**	**WT**	**WT**	**65**	**M**	**6**	**na**
**P52**	**autonomous cortisol secretion**	**WT**	**R201C**	**43**	**F**	**2,8**	**19,5**
**P53**	**autonomous cortisol secretion**	**WT**	**WT**	**65**	**M**	**4**	**3,9**
**P54**	**autonomous cortisol secretion**	**WT**	**WT**	**47**	**M**	**2,5**	**na**
**P57**	**autonomous cortisol secretion**	**WT**	**WT**	**61**	**M**	**4,8**	**2,9**
				46,5	27F/ 7M	4,0	13,1

Supplementary Table S2: Clinical characteristics of all adrenocortical tumours other than CPA used. Median age and mean values are indicated after each tumour group. Tumour sizes are given in cm, cortisol post dexamethasone results are given in µg/dl. ARQ: aldosterone-renin quotient.

#	Diagnose	comment	Age	Sex	Tumor size	Cort post Dexa	ARQ	Aldo
P59	EIA		33	M	4	na	na	na
P60	EIA		59	M	3	na	na	na
P61	EIA		53	F	2,8	0,9	na	na
P62	EIA		69	M	6	na	na	na
P63	EIA		49	F	5	1,3	na	na
P64	EIA		47	F	6,1	na	na	na
P65	EIA		55	F	6,5	13,1	na	na
P66	EIA		54	M	5,4	1,2	na	82,7
P67	EIA		51	M	8,5	1,4	na	na
P68	EIA		67	F	3,4	na	16,9	42,2
P69	EIA		72	M	3,3	2,3	3,1	82,2
P70	EIA		49	M	7	na	na	na
P71	EIA		63	M	4	2,7	na	na
P72	EIA		45	M	2	na	na	na
P73	EIA		53	M	4	1,8	na	na
P74	EIA		50	M	4	2,2	na	na
P75	EIA		56	M	4,7	1,1	na	na
P76	EIA		47	F	1,3	na	na	na
P77	EIA		60	M	1	na	na	na
P78	EIA		80	M	4	na	na	na
P79	EIA		43	M	4,5	na	na	na
P80	EIA		60	F	1,3	na	na	na
P81	EIA		66	F	2,4	1,3	na	na
P82	EIA		48	M	1	na	na	na
P83	EIA		82	F	5	na	na	na
			54	9F /16M	4,0	2,7		

#	Diagnose	comment	Age	Sex	Tumor size	Cort post Dexa	ARQ	Aldo
P84	APA		44	F	1,3	na	na	na
P85	APA		42	F	0,9		31,2	256
P86	APA		51	F	2	na	58	200
P87	APA		36	F	1,5	na	na	469
P88	APA		46	M	1,6	na	na	102
P89	APA		34	F	1,6	na	na	288
P90	APA		52	M	0,9	na	73,0	na
P91	APA		54	F	1,7	na	na	na
P92	APA		58	F	2,1	na	na	201
P93	APA		60	F	0,9	na	na	246

P94	APA		51	F	3,8	na	na	668
P95	APA		60	M	0,4	na	455	237
P96	APA		59	M	1	na	na	na
P97	APA		53	F	1,8	na	na	130
P98	APA		41	F	1,4	na	128	320
P99	APA		46	F	3	na	na	562
P100	APA		61	F	1,8	na	198	495
P101	APA		36	F	1,6	na	na	na
P102	APA		49	F	3,3	1,9	na	na
P103	APA		52	M	1,5	na	na	na
			51	15F/ 5M	1,7		157,2	321,1

#	Diagnose	secretion	Age	Sex	Tumor size	ENSAT stage	Ki67%	Weiss score
P104	ACC	androgen	66	F	8,2	na	10	3
P105	ACC	cort	77	F	3	3	15	na
P106	ACC	cort+andr	49	F	15	3	10	7
P107	ACC	cort	46	F	9	2	20	6
P108	ACC	cort	46	M	9	2	na	5
P109	ACC	cort+andr	42	F	11	2	10	5
P110	ACC	cort+andr	55	F	13	4	na	8
P111	ACC	cort	51	F	8,5	3	2	4
P112	ACC	cort	38	F	9	2	30	5
P113	ACC	na	43	M	15	4	20	7
P114	ACC	inactive	47	F	24	3	15	7
P115	ACC	na	50	F	13	2	na	na
P116	ACC	cort+andr	72	M	14	2	10	6
P117	ACC	cort+andr	79	F	6,5	3	10	5
P118	ACC	inactive	46	M	6,5	2	3	3
P119	ACC	cort+andr	75	F	6,5	4	20	7
P120	ACC	androgen	60	M	12,5	2	10	7
P121	ACC	cort+andr	43	F	17	4	10	4
P122	ACC	androgen	53	F	5,1	2	15	4
P123	ACC	cort+andr	57	F	15	4	15	na
P124	ACC	na	58	F	8	2	2	4
P125	ACC	inactive	80	F	8,2	3	10	na
P126	ACC	cort	66	F	7	3	na	5
P127	ACC	cort+aldo	34	F	16	2	40	8
P128	ACC	na	43	F	13,5	3	70	9
P129	ACC	na	57	M	19	2	30	9
P130	ACC	na	na	F	na	na	na	na
P131	ACC	inactive	49	F	11	2	30	7
P132	ACC	na	67	M	9	2	2	7
P133	ACC	cort+andr	58	F	7,5	4	na	5
P134	ACC	cort+andr	66	M	13	4	10	7

P135	ACC	cort	52	F	8	2	20	na
P136	ACC	cort+andr	36	M	19	3	20	8
			53	24F/ 9M	11,3		17	6
